# Analysis of Time to Event Outcomes in Randomized Controlled Trials by Generalized Additive Models

**DOI:** 10.1371/journal.pone.0123784

**Published:** 2015-04-23

**Authors:** Christos Argyropoulos, Mark L. Unruh

**Affiliations:** Department of Internal Medicine, Division of Nephrology, University of New Mexico, Albuquerque, New Mexico, United States of America; Sudbury Regional Hospital, CANADA

## Abstract

**Background:**

Randomized Controlled Trials almost invariably utilize the hazard ratio calculated with a Cox proportional hazard model as a treatment efficacy measure. Despite the widespread adoption of HRs, these provide a limited understanding of the treatment effect and may even provide a biased estimate when the assumption of proportional hazards in the Cox model is not verified by the trial data. Additional treatment effect measures on the survival probability or the time scale may be used to supplement HRs but a framework for the simultaneous generation of these measures is lacking.

**Methods:**

By splitting follow-up time at the nodes of a Gauss Lobatto numerical quadrature rule, techniques for Poisson Generalized Additive Models (PGAM) can be adopted for flexible hazard modeling. Straightforward simulation post-estimation transforms PGAM estimates for the log hazard into estimates of the survival function. These in turn were used to calculate relative and absolute risks or even differences in restricted mean survival time between treatment arms. We illustrate our approach with extensive simulations and in two trials: IPASS (in which the proportionality of hazards was violated) and HEMO a long duration study conducted under evolving standards of care on a heterogeneous patient population.

**Findings:**

PGAM can generate estimates of the survival function and the hazard ratio that are essentially identical to those obtained by Kaplan Meier curve analysis and the Cox model. PGAMs can simultaneously provide multiple measures of treatment efficacy after a single data pass. Furthermore, supported unadjusted (overall treatment effect) but also subgroup and adjusted analyses, while incorporating multiple time scales and accounting for non-proportional hazards in survival data.

**Conclusions:**

By augmenting the HR conventionally reported, PGAMs have the potential to support the inferential goals of multiple stakeholders involved in the evaluation and appraisal of clinical trial results under proportional and non-proportional hazards.

## Introduction

The Cox Proportional Hazard (CPH) model, proposed in 1972[1] is currently the preferred method for the analysis of censored data from randomized controlled trials (RCTs) and observational studies of time to event outcomes. CPH circumvents the statistical complications created by censoring of survival data, by working in the probability rather than the time domain and calculates Hazard Ratios (HR) as measures of treatment efficacy. Despite the popularity of HR among clinicians, the endorsement by the Cochrane Handbook for systematic reviews[2] and a CONSORT statement that recommends them as measures of intervention efficacy[3], HRs are not ideal summaries of intervention efficacy for a number of reasons. *First*, they are routinely misinterpreted by clinicians[4] as relative risks, odds ratios or as relative speed. *Second*, HRs do not readily convey the information required by patients (absolute measures of survival) or third parties in the context of shared decision making[5,6] and health care evaluation. *Lastly*, the HRs are sensitive to the proportionality assumption of the PH model; when the latter is violated, the estimated HRs no longer provide unbiased summaries of the treatment effect as the true HR varies with time. These potential pitfalls are addressable by shifting to parametric regression models, direct modeling of the survival[7], or the hazard function with flexible functions of time.

In this paper, we show how flexible hazard models may be used as a comprehensive analytical framework for the simultaneous generation of multiple treatment effect measures under proportional or non-proportional hazards. The approach we propose borrows concepts from lifetable analysis[1,8,9] and techniques from Generalized Additive Models(GAM) [10] for Poisson regression (PGAM) to achieve these goals. We undertake extensive numerical evaluations and statistical simulations to demonstrate that the PGAM approach can yield unbiased estimates of treatment effects and survival probabilities, comparable to the Cox model and the Kaplan Meier (KM) procedure, under proportional and non-proportional hazards. We illustrate the approach in two clinical datasets: a two arm oncology clinical trial (IPASS)[11] for which we reconstruct the data from the study manuscript, and a nephrology RCT (HEMO)[12] for which patient level information is available, thus enabling a covariate adjusted analysis. IPASS is an example of a study in which the violation of the proportionality assumption complicates the interpretation of the intervention effect. HEMO is a high-quality, RCT of long duration conducted under evolving standards of care on a heterogeneous patient population. Natural questions to ask in such a situation, is whether the estimate of the HR may be confounded by secular trends in treatment standards or center effects, and whether the treatment effect was the same across patient subgroups. We consider the implications of the proposed approach for the reporting and communication of different treatment effect measures to clinical audiences and policy makers and the utility of PGAMs to support objective trial analyses that cater to the inferential needs of different consumers of clinical trial data.

## Materials and Methods

### Survival analysis with Gaussian quadrature and Poisson Generalized Additive Models

By splitting the observation time in arbitrary small intervals (the "lifetable" approach), previous work has shown that it is possible to use Poisson regression approximations for survival analysis (see S1 Appendix, Section A1 for a timeline of these ideas and a literature survey). Our approach extends previous work by proposing a time-splitting scheme based on Gaussian quadrature that limits the computational resources required to apply this method, while maintaining control over the numerical precision of the approximation. Within this framework we utilize flexible models (e.g. with within Poisson regression (Poisson Generalized Additive Models—PGAM) to model the log-hazard function. The recent availability of *R* software packages[10] for the fast fitting of GAMs to big datasets makes this approach practical for large applications. As we demonstrate below, PGAMs are ideally suited for clinical trials as they support a variety of analyses relevant to the context of RCTs: non-proportional hazards modeling, stratification variables, correlated outcomes, treatment by covariate interactions and multiple time scales. Non-linear functions of PGAM estimates (PGAM predictions) and their confidence intervals[13] may be used as alternative to the hazard ratio measures of treatment effects (S1 Appendix, Section A2).

### Approximating Survival Likelihoods with Poisson Models

Consider a set of *N* individual patients for whom individual observations are available at times $F={\left\{F_{i}\right\}}_{i=1}^{N}$ with censoring indicators $D={\left\{\delta_{i}\right\}}_{i=1}^{N}$ assuming the value of zero if the corresponding observation was censored (patient had not experienced the event of interest) and one otherwise. Patients are assumed to come under observation at times $Ε={\left\{E_{i}\right\}}_{i=1}^{N}$ and these times may not necessarily be equal to zero in order to allow for delayed entry into the study. Under the assumption of non-informative censoring, the likelihood is given by:

$$\prod_{i=1}^{N}\frac{f{\left(F_{i}\right)}^{\delta_{i}}\times S{\left(F_{i}\right)}^{1-\delta_{i}}}{S(E_{i})}=\prod_{i=1}^{N}h{\left(F_{i}\right)}^{\delta_{i}}\times \exp(-\int_{E_{i}}^{F_{i}}h(t)dt)$$ Eq 1

where in going from the left to the right hand side we have use of the definitions of the hazard, cumulative hazard survival, density functions. Numerical integration (quadrature) methods may be used in order to approximate the integral with a weighted sum over a set of nodes *t*_*i*,*j*_:

$$\int_{E_{i}}^{F_{i}}h(t)dt≅\sum_{j=1}^{n_{i}}w_{i,j}h(t_{i,j})$$ Eq 2

After substituting the sum for the integral and introducing the auxiliary variables *d*_*i*,*j*_ = 1 if the *nodes* of the quadrature (*t*_*i*,*j*_) are event times and zero otherwise, we obtain the(approximate) likelihood:

$$\prod_{i=1}^{N}\prod_{j=1}^{n_{i}}h{\left(t_{i,j}\right)}^{d_{i,j}}\times e^{-w_{i,j}h(t_{i,j})}$$ Eq 3

This can be recognized as the kernel of the Poisson likelihood with variable exposures (offsets) given by the logarithm of the quadrature weights (*w*_*i*,*j*_). To use this connection in applications, one expands the dataset with additional “pseudo-observations” for each individual. These are inserted at the set of nodes of the integration scheme other than the event times. Parameter estimates and covariance matrices produced by the maximization of the Poisson likelihood are valid approximations to analogous functions obtained by optimizing the exact likelihood in a well defined sense: as the number of nodes of the integration scheme increases, the accuracy of the discrete sum approximation improves, so that in the limit *n*_*i*_ *→∞* the approximation becomes exact. For a finite number of nodes, *n*, the ratio of the approximate and the exact likelihoods depends on the error term, *R*_*n*_ *(F*_*i*_,*E*_*i*_ *;f)* of the quadrature rule. The total error incurred by the *N* numerical integrations in the approximate likelihood is bound by:

$$\prod_{i=1}^{N}e^{R_{n}(F_{i},E_{i};f)}=e^{N\langle R_{n}(F_{i},E_{i};f)\rangle }\leq N\times \underset{i}{\max}\left(e^{R_{n}(F_{i},E_{i};f)}\right)$$ Eq 4

The accuracy of the Poisson approximation improves exponentially fast with the error of the quadrature rule. Consequently judicious use of the quadrature rule may be used to achieve a balance between numerical accuracy and computational resources (number of nodes/size of the dataset) used during model fitting. We consider these issues in the following sections.

### Time Discretization Schemes for the Analysis of Survival Data

The simplest quadrature rule for the integration of the hazard function is the *trapezoid* rule, which splits the dataset to the unique failure times (as in the approach for grouped survival data by Efron[8] and implicitly in the PH[1]) or even the unique observation times irrespective of censoring[14–16]. This discretization expands the original dataset to very large sizes equal to 0.5×(*N* ^*2*^ *+N*) i.e. >500000 Poisson-like observations for a study of *N =* 1000 people. To overcome this computational limitation, we applied the Gauss-Lobatto (GL) quadrature rule, in which the set of nodes consists of the endpoints augmented by additional abscissas symmetrically distributed around zero in the interval [–1,1] (see[17] Section 4.6.1). Gaussian quadrature converges exponentially fast and thus requires fewer nodes to achieve the same level of precision (see[17] pages 179–193) as the trapezoidal rule. The practical implication of using the GL rule as opposed to other Gauss rules[18] is that the entry and last follow-up time are used as nodes. This feature introduces two constraints into the Poisson approximation: a) that no patient can fail at their entry time (an implicit assumption to all survival modeling) and b) that time/event status at the end of follow-up are represented exactly in the dataset.

The number of the nodes can be heuristically selected by considering the functional form of the error (remainder) term in the GL rule:

$$R_{n}(F_{i},E_{i};f)=\underset{A}{\underset{︸}{-\frac{n{\left(n-1\right)}^{3}{\left[\left(n-2\right)!\right]}^{4}}{\left(2n-1\right){\left[\left(2n-2\right)!\right]}^{3}}}}\overset{B}{\overset{︷}{{\left(F_{i}-E_{i}\right)}^{2n-1}}}f^{\left(2n-2\right)}(\xi ),\;E_{i}<\xi <F_{i}$$ Eq 5

This expression, which follows from transforming the error of the rule in the [–1,1] interval (page 104[19]) to the domain of integration [*E*_*i*_,*F*_*i*_], shows that the absolute magnitude of the error depends on a term (*A*) that decreases very fast with the order of the integration, a factor that increases with the length of the integration interval (*B*) and finally the value of the *2n-2th* order derivative of the hazard function within the domain of integration. The kind of functions we will consider in this work, model the log-hazard as cubic or one-dimensional thin plate splines, both of which are local third degree polynomials. Consequently, the *2n-2th* derivative of the hazard at any given point is given by:

$$f^{\left(2n-2\right)}(\xi )={\left(\exp(\gamma +a\xi +b\xi^{2}+c\xi^{3})\right)}^{\left(2n-2\right)}=\underset{h(\xi )}{\underset{︸}{\exp(\gamma +a\xi +b\xi^{2}+c\xi^{3})}}Q(\xi )\underset{n}{\sim }h(\xi )\xi^{4n-4}{\left(3c\right)}^{2n-2}$$ Eq 6

This expression which follows from repeated application of the chain rule, shows that as more nodes are added, the last term in Eq 5, which is the product of a high order polynomial (*Q*(*ξ*)) and the hazard function, will asymptotically increase in absolute magnitude. Hence, there are diminishing returns to be expected from increasing the number of nodes after some point, suggesting that a limited number of nodes will suffice for applications.

### Flexible (log-)hazard modeling in randomized trials

The building block for all analyses presented in this paper resolves the (log-) hazard function at of the *i* ^*th*^ patient at the *j* ^*th*^ time point as:

$$\log(h(t_{i,j}))=\lambda_{0}(t_{i,j})+x_{i}\beta$$ Eq 7

In the previous expression *λ*_0_(*t*) is the baseline log-hazard, **x**_**i**_ are treatment group assignments and possibly, but not necessarily, other baseline covariates which one wants to adjust the analysis for, and **β** are the corresponding log-hazard ratios. This model can be extended to accommodate the following analyses:

- *Stratification*, which substitutes the single baseline log-hazard with additional smooth functions of time, e.g. in a multicenter study one can specify one such function for each center:

$$\log(h(t_{i,j}))=\sum_{k=1}^{S}\lambda_{k}(t_{i,j})+x_{i}\beta$$ Eq 8

- *Non-proportional effects*, in which the proportionality assumption is relaxed for one or more covariates and the model is augmented with the interactions of these covariates with time. This is conceptually and mathematically similar to carrying out a stratified analysis if the aforementioned covariate is categorical (e.g. gender) and to a varying coefficient model if the covariate is continuous (e.g. age). In the context of a randomized trial one can use this feature to simultaneously detect and account for the violation of the proportionality assumption for the treatment effects. This adjustment can take one of two equivalent forms, which for a two arm trial may be equivalently expressed as:

$$\log(h(t_{i,j}))=\lambda_{0}(t_{i,j})+x_{i}\beta +\beta_{T}(t)=\lambda_{0}(t_{i,j})+x_{i}\beta +\beta_{T}+\beta_{T}^{NC}(t)$$ Eq 9

with $\beta_{T}(t)=\beta_{T}+\beta_{T}^{NC}(t)$, the overall treatment effect, *β*_*T*_ the component of the log-hazard ratio that is constant with time and $\beta_{T}^{NC}(t)$ the non-constant, time varying effect. A statistical test for the equality of $\beta_{T}^{NC}(t)$ to zero amounts to a test for the proportionality of hazards, while a test for the equality of *β*_*T*_ to zero is simply a test for the constant component of the log-hazard similar to the one conducted by the PH model. Finally, testing whether *β*_*T*_(*t*)is equal to zero is a test for an overall difference of the hazard function, and thus the survival, between treatment arms or more generally for different covariate values.

- *Heterogeneous/correlated/frailty* effects obtained by augmenting the second component of the log-hazard function with a random effects model to model correlations among individuals within the same cluster (*k)*.

$$\log(h(t_{k,i,j}))=\lambda_{0}(t_{i,j})+x_{i}\beta +z_{k}b,\;b\sim N(0,G)$$ Eq 10

In the equation above the covariates for the random effects component are given by **z**_**k**_ and the random effects themselves by **b**. The latter are assumed to be normally distributed with a covariance matrix equal to **G**. For example in a multicenter study one could assume that the treatment effect in the various study centers are not identical, but are related e.g. as realizations from a hypothetical population of centers with a common mean and (co-)variance matrix. Cluster randomized trials (CRT), in which whole centers rather than individual patients are randomized to specific interventions, are a special case of this formulation.

- *Multiplicity of time scales*, in which the effects of additional scales are modeled with smooth functions of the corresponding time scale. RCTs are conventionally analyzed under the assumption that the only time-scale that is relevant is “study time”, and thus concentrate on the analysis of the duration of the follow-up. The incorporation of *disease scale* effects relaxes the assumption that the intervention has the same effects in participants at different points during their disease process. Allowing for *period* effects, i.e. those occurring on the *calendar time* scale (“secular trends”) acknowledges the possibility that changing standards of therapy may have impacted the effectiveness of the intervention. Even though the randomized nature of clinical trials is thought to protect against secular trends as they would affect all trial arms, it cannot guard against trends that affect preferentially the intervention arm. Hence, one could model the hazard not only as function of the time since the beginning of observation, but also as a function of the calendar time the observation was made:

$$\log(h(t_{i,j},c))=\lambda_{0}(t_{i,j})+x_{i}\beta +f(c)$$ Eq 11

Explicit modeling of interactions among multiple time scales is implemented through multidimensional (tensor-product) smooth functions that adjust the baseline hazard function.

### Flexible log-hazard penalized fitting in Poisson Generalized Additive Models

In the PGAM framework the log-hazard model (Eq 7) and its extensions (Eqs 8–11), a smooth function is decomposed as a linear combination of a finite number of basis functions:

$$f(x)=\sum_{j=1}^{J}g_{j}(x)\theta_{j}$$ Eq 12

Associated with this representation is a “wiggliness” measure of the function’s smoothness, *J*(*f*) = **θ^T^Sθ**, where **S** is a symmetric positive *semi-definite matrix* of coefficients. This matrix penalizes the wiggly components of *f* but leaves the remaining unpenalized. Common choices for the basis functions include cubic smoothing or thin plate splines over a small set of a number of knots[10]. In this work we fit such spline models using only a small number of degrees of freedom, corresponding to a few knots (5–10) that are placed in corresponding quantiles of the partitioned observation time. The PGAM formulation may be expressed as a generalized mixed effects model (see Chapter 6 in [10], particularly pages 316–318 and section 3.19 in[20]) through the eigen-decomposition of the **S** matrix into components that are unpenalized (fixed effects) and those that are penalized (random effects). For the cubic spline basis the former would include the constant and linear term, while the quadratic and cubic factors would be penalized. The one-dimensional thin plate spline which is a local cubic polynomial[10] without a quadratic term, also penalizes the cubic term but not the constant or the linear function. Irrespective of the choice of the basis, *penalization* of the higher order components not only results in smooth, parsimonious models but also *has the added benefit of improving the approximation error due to the numerical quadrature*. The order of the latter depends on the coefficient of the cubic factor (Eq 6), so that penalized fitting, that shrinks the value of this coefficient towards zero, moderates the magnitude of the error.

Fitting of GAMs may be undertaken via Generalized Cross Validation (GCV), or exploiting their mixed model representation with Restricted Maximum Likelihood (REML). We collectively designate the components of the smooths (*θ*_*j*_ *)*, the constant terms (**β**), the frailty random effects (**b**) as $\hat{\psi }$, and the associated variance-covariance matrix as ${\hat{V}}_{\psi }$. By specifying a *prediction matrix* X_p_, i.e. a combination of covariates for which one wants to obtain predictions about the log-hazard function, one can use the multivariate normal distribution $N(\hat{\psi },{\hat{V}}_{\psi })$ to generate predictions and their standard errors. For linear functions of the fitted model, e.g. the difference in the log-hazard function between the groups of a two-arm RCT, linear contrasts and standard probability theory (see page 245 in [10]) are sufficient. For *non-linear* functions of the fitted model, e.g. alternative to the HR measures of treatment effect, a post-estimation simulation is employed to generate predictions and their associated standard errors instead. This involves sampling of a large number of replicates ${\tilde{\psi }}_{k}$ from $N(\hat{\psi },{\hat{V}}_{\psi })$, calculating the non linear function implied by the prediction matrix X_p_ for each set of replicate values and summarizing the simulations of the non-linear functions in terms of means, standard deviations and quantiles. In S1 Appendix, Section A3 we highlight the simulation steps required to predict the survival probability.

### Unconditional, Conditional and Population Averaged PGAM Treatment Effects

Treatment effects may be calculated from a *unconditional* perspective in which the mean outcome is computed for intervention and control group, averaging over the entire population in the study without adjusting for any covariates[21]. Though this is the most commonly employed method in the clinical literature, a substantial number of RCTs employ adjusted analyses and we consider them as well. To calculate these adjusted values we employ the following procedure: after fitting an adjusted flexible hazard model, we use this model to generate predictions for each individual in the study, which are then averaged over all the participants allocated to a given arm. This approach, known as the *corrected group prognosis*[22] for survival probability predictions, has been shown to generate *directly adjusted survival probabilities*[23–25] that closely track the unadjusted, KM curves in real world studies[22]. The interventions are subsequently compared taking groupwise averages over the distribution of the covariate values in the study participants and then forming differences or ratios depending on the effect measure chosen.

### Simulations

We undertook simulations to evaluate the error of the time discretization implied by the GL rule and the performance of the PGAM estimates vis-à-vis those generated by the Cox model (HR) and the KM procedure for the survival function. Finally we assessed whether the PGAM can yield unbiased estimates of the RMST as an alternative to HR, in the setting of either proportional or non-proportional hazards. The latter simulations are informative about the robustness of the PGAM with respect to mis-specification: unless one knows exactly the form of the hazard rate function, almost any basis function will be locally mis-specified against the truth. For example, if the deviation from the baseline hazard obeys the proportional hazards assumption, then the cubic or a thin plate spline third degree PGAM basis is a mis-specified model. One would like to know whether mis-specified PGAMs can still yield unbiased estimates of the treatment effect on the RMST scale that does not depend on the proportional hazards assumption. For each of the simulations described below, we used distinct and widely spaced seeds for the random number generator to reduce correlations between the datasets generated. We used the same set of datasets when comparing the PGAM against the HR or the KM. This “matched pair design” eliminates the within sample variability and increases the sensitivity of the simulation to detect differences between the statistical methods compared[26].

### Numerical Evaluation of the Gauss Lobatto Error

A numerical exploration of the error associated with the use of the GL rule may be undertaken by considering bounds of Eq 5. Since cubic and thin plate splines are local cubic polynomials, our investigations focused on error incurred during integration of exponentiated cubic polynomials. In particular, the direct optimization of the *2n-2th* derivative of the hazard function for various combinations of the parameters (*γ*, *a*, *b*, *c*) of the polynomial is used to bound this error for a given length of the integration interval (*F*_*i*_ *-E*_*i*_). Combinations of parameters of the cubic polynomial were generated as *quasi random* numbers[27,28] using a Sobol *low discrepancy* sequence[29]. These low discrepancy numbers have the property to cover the multidimensional space of the parameters of interest more uniformly than pure (pseudo-) random numbers. Numbers generated in the unit hypercube were then mapped to the space [-1,1]^3^×[0,1] defining the range of the parameters (*γ*, *a*, *b*, *c*) respectively. We selected this range to yield a range of expected survival times typical of many clinical trials (roughly 3.2–44.1 months, median 8.9, mean 10.5 months) in the fields of oncology and nephrology. For each unique combination of (*γ*, *a*, *b*, *c*) we calculated the mean (expected) survival time by numerical integration (MST) as the area under the curve of the corresponding survival function (S1 Appendix, Section A2). Subsequently we substituted the calculated MST for *F*_*i*_ into Eq 5 and maximized this expression over *ξ* assuming right censored data (i.e. *E*_*i*_ = 0). Such a maximization yields the worse-case error for the *average* lifetime of the survival distribution corresponding to a particular choice of (*γ*, *a*, *b*, *c*). An arithmetic average yields an estimate of the expected error of the GL rule over the space (*γ*, *a*, *b*, *c*) for a given order of integration (*n* in Eq 5). We used 1000 pseudo random points to calculate this average error which is a typical number of quasi-random points for the low (four-) dimensional space we explored.

### PGAM estimates against the Kaplan Meier estimator and the Cox model

We simulated survival from the three different parametric lifetimes: Weibull, Gompertz and Lognormal. We assumed four different baselines from each of these distribution parameterized as: a) *Weibull* (shape/scale: 0.8/0.1, 1.2/0.1, 0.8/0.2, 1.2/0.2) b) Gompertz (shape/scale: -0.01/0.05, 0.1/0.05,-0.01/0.15, 0.1/0.15) and c) Lognormal (mean/scale: 0.5/1.0, 1.0/1.0, 1.5/1.8, 1.5/0.8). Within each of the three parametric families, these choices lead to survival curves that are well separated from each other over time. To assess the performance of the PGAM against the Kaplan Meier estimator we simulated 300 individuals from each of the 4 aforementioned Weibull, Gompertz and Lognormal baselines under two different percentages (30% and 70%) of censored observations. Censoring was assumed to follow an exponential distribution with a rate parameter that was adjusted using numerical integration/optimization to yield a censoring percentage of either 30% or 70% for each simulation scenario. The resulting 12000 datasets were analyzed with the PGAM and the KM procedure and survival probability estimates were generated at 50% and 95% of the maximum observation time for each dataset. We selected these points in order to compare methods at points in time with a sufficient number of events to calculate the survival probability and its associated standard errors with the KM procedure. For the PGAM, these survival probabilities are obtained as predictions using the approach described in S1 Appendix, Section A3. The number of the repetitions was selected to yield an estimate of the survival probability to an accuracy (*δ*) of slightly better than 9% of its standard deviation (*σ*) according to the formula[26]:$B={\left(\frac{Z_{1-a/2}\sigma }{\delta }\right)}^{2}$, with *Z*_*1-a/2*_the 1-*a*/2 quantile of the standard normal distribution.

To assess the ability of the PGAM to yield unbiased estimates of the HR, we simulated studies of 600 participants (300 per arm) with the survival of the control arm being given by each of the 4 aforementioned Weibull and Gompertz baselines. Within each of these simulations the survival of the experimental arm was assumed to follow the same parametric baseline but with a HR of 0.5, 0.7, 0.9 for each of the baselines assessed. For these simulations we also assumed exponential censoring under two different censoring percentages i.e. 30% and 70%. Therefore our simulation strategy for assessing the PGAM evaluated a total of 2 x 4 x 3 x 2 = 48 unique combinations of parametric families/baseline hazards/hazard ratios and censoring proportions. We simulated 500 repetitions of trials from each combination (a total of 24000 datasets) in order to yield estimates of the HR with an accuracy that was slightly better than 9%. These datasets were analyzed with both the Cox proportional hazards model (as implemented in the *coxph* package in R) and the PGAM approach and the corresponding hazard ratio point estimates was and standard errors were stored for subsequent analyses (see 2.4).

### PGAM estimates of the RMST

For the evaluation of the PGAM to yield unbiased estimates of the RMST under either proportional or non-proportional hazards we simulated from four different lifetime distributions that were implicitly defined through their baseline log-hazard function:

$$\begin{array}{c} \log(0.05)+0.20t+0.02t^{2}\text{, (A)}\\ \log(0.10)-0.10t+0.02t^{2}\text{, (B)}\\ \log(0.05)-0.02t+0.02t^{2}\text{, (C)}\\ \log(0.10)+0.10t+0.01t^{2}\text{, (D)} \end{array}$$

Similar to the HR we assumed randomized trials of 600 patients (300 in each arm), with the log-hazard function of the control given by A-D and that of the experimental arm defined through proportional (PH), linear, quad(ratic) and linear-quadratic (LQ) deviations from the baseline log hazard:

$$\begin{array}{c} \log(HR)\text{, (PH)}\\ \log(HR)+at\text{, (Linear)}\\ \log(HR)+bt^{2}\text{, (Quad)}\\ \log(HR)+at+bt^{2}\text{, (LQ)} \end{array}$$

We examined two different censoring proportions (30 and 70%), giving a total of 32 combinations of baseline hazards/deviations from the hazard and censoring percentages. For each of these 32 unique combinations of two arm trials we generated 500 datasets, sampling (*HR*, *a*, *b*) uniformly over the domain: [log(0.7),log(0.9)]×[-0.1,0.1]×[-0.009,0.009]. By varying these parameters in the 16000 datasets rather than keeping them fixed, we were able to assess the PGAM performance against a large combination of treatment effects and deviations (or near deviations) from non-proportionality. With these particular choices for the baseline hazard and deviation from baseline we ensured that the PGAM will be mis-specified with respect to the simulated truth. In particular, the cubic spline basis we utilized is locally a third degree polynomial and it is only through shrinkage of the quadratic and cubic coefficients that these may approximate the Linear and LQ deviations. As the linear and the constant coefficients are not penalized by the PGAMs, the latter are always mis-specified against the PH and the Quad deviations from the baseline. To generate individual lifetimes within each synthetic dataset we employed the Bender, Augustin and Blettner algorithm for simulating from general lifetime distributions defined through their baseline hazard[30]. This is a computationally demanding algorithm that combines numerical integration and non-linear root solving to transform uniformly distributed variates over the unit interval to lifetimes from the desired survival distribution. For the purpose of this paper we implemented the algorithm in the *R* programming language using the builtin numerical integration and root finding capabilities of base *R*.

To calculate the RMST, the PGAM was applied to generate estimates of the baseline hazard function in the control arm and the deviation from baseline in the experimental arm. Using the approach described in S1 Appendix, Section A3, we generated survival estimates at times given by the nodes of a 10 node GL rule defined in the interval [0,*T*_*max*_] with *T*_*max*_ equal to the maximum follow up time in each survival dataset. These were weighted by the corresponding weights of the GL rule so as to calculate the area under the survival curve of the control and experimental arms. The two areas were then subtracted to generate a single estimate for the RMST; the average and the standard error of these differences over the number of simulations were stored for subsequent analyses. For each dataset we calculated the theoretical RMST from the simulated parameters and baseline hazards with the adaptive numerical integrator (21 point Gauss Kronrod[31]) built in the *R* language.

### Performance measures for evaluating simulation output

We used the simulations to assess the PGAM the Cox and the Kaplan Meier curve in terms of bias, accuracy and coverage. Each simulation generates an estimate for a parameter of interest ${\hat{\beta }}_{i}$(e.g. log-hazard ratio, survival probability or RMST); the standard error of ${\hat{\beta }}_{i}$ over all simulations,$SE({\hat{\beta }}_{i})$, is an empirical estimate that can be used to index the absolute bias. We chose to report this *standardized bias* rather than the absolute bias, since the consequences of the bias depend on the uncertainty of the parameter of interest[26]. We used the Mean Square Error (MSE) as an index of *accuracy*. Finally we report two measures of coverage: *p-value coverage*, i.e. the proportion of times the 95% confidence interval includes the true value of the parameter of interest and the *average length of the confidence interval* (*CIL*) over the simulations performed. P-value coverage was considered acceptable if the p-values from a set of *N* simulations fall within 2 Standard Errors of the nominal coverage probability (*p*)[26],$SE(p)=\sqrt{p(1-p)/N}$. To the extent that the parameters of interest are unbiased, then narrower confidence intervals imply more precise estimates[26]. These in turn may translate in higher efficiency and power. Formulas for the calculation of the standardized bias, MSE, p-value and confidence interval length may be found in Table 1 of Burton et al[26].

**Table 1:** Estimates of the Hazard Ratio and the upper (UCI) and lower (LCI) confidence intervals of the treatment effect of Gefitinib in the IPASS trial.

	HR	LCI	UCI
Cox	0.73	0.64	0.83
GL3	0.68	0.59	0.77
GL4	0.77	0.67	0.88
GL5	0.72	0.64	0.82
GL6	0.72	0.63	0.82
GL7	0.73	0.64	0.83
GL8	0.73	0.64	0.83
GL9	0.73	0.64	0.83
GL10	0.73	0.64	0.83
GL11	0.73	0.64	0.83
GL12	0.73	0.64	0.83
GL13	0.73	0.64	0.83
GL14	0.73	0.64	0.83
GL15	0.73	0.64	0.83
GL16	0.73	0.64	0.83
GL17	0.73	0.64	0.83
GL18	0.73	0.64	0.83
GL19	0.73	0.64	0.83
GL20	0.73	0.64	0.83

Cox: estimate based on a Cox proportional hazard model. GL: Gauss Lobatto rule. The number behind GL designates the number of nodes in the numerical integration rule. This number is equal to the number of sub-intervals used to split each observation time in the dataset.

### Clinical trial datasets

We used data from two large RCTs: IPASS and HEMO to illustrate the utility of PGAM to provide unbiased estimate of treatment effects under non-proportional hazards and multivariable adjustments respectively. The IPASS[11] trial was a randomized controlled trial reporting on the progression-free survival of two regimens of gefitinib v.s. carboplatin-paclitaxel in patients with advanced pulmonary adenocarcinoma. The primary outcome was analyzed using an unadjusted proportional hazards model. The study’s results were reported with this model despite the violation of the proportional hazards assumption implied by the crossing of the two survival curves. Since we did not have access to the individual participant data (IPD), we reconstructed the dataset from the Kaplan Meier curves appearing in the published study manuscript. Reconstruction of IPD is not an integral component of our methodology; we merely used it to obtain access to a real world dataset that violates the proportionality assumption in a way that limits the clinical inferences from the particular study [32,33]. Reconstructing IPD from the published Kaplan Meier curves is a well established approach for secondary analyses of survival data (including meta-analysis) when the actual IPD are not accessible[34–38]. To reconstruct the IPD from the IPASS trial, we digitized the survival curves from the images in the Portable Document Format (PDF) version of the manuscript with the *Engauge* open source software[39]. Subsequently, we use the digitized curves along with reported number of patients at risk to reconstruct the study data with the aid of a recently reported algorithm[40]. The reconstructed individual patient data (provided in S1 and S2 Datasets) are then analyzed with PGAMs. R source code for the analysis of the reconstructed IPASS dataset (including the generation of survival curves and the calculation of all measures of treatment efficacy considered in this paper) via PGAMs is provided in S1 Text.

The HEMODIALYSIS (HEMO) study[12] was a multicenter RCT that used a 2 x 2 factorial design to examine the effects of HD dose and membrane flux on survival in *prevalent* hemodialysis patients (those who had been on dialysis for more than three months, with minimal residual kidney function). The primary outcome of the study was all cause mortality with the HR chosen as measure of efficacy. In many aspects HEMO is an ideal dataset for the evaluation of PGAMs. First, the statistical analysis protocol of the primary investigators[41] specified the covariates to be used for the analysis of outcomes, allowing us to verify the results of PGAMs against those of the Cox model. Second, the known variability of dialysis practices and outcomes across dialysis clinics and centers[42] provides an opportunity to explore center heterogeneity with respect to the baseline hazard in stratified analyses.

During the 6.5 years of the HEMO study (May 1995—December 2001) there was continuous improvements in the mortality rate of prevalent hemodialysis patients in the United States (Figure 5.1 in [43]) and the performance characteristics of the dialysis membranes. Hence asking whether these secular trends had an impact in the assessment of study outcomes is a valid question. Hence, we undertook a multiple time scale modeling of the outcomes in HEMO in the three scales of study time, disease duration and calendar time.

The PGAM re-analysis of HEMO affords the opportunity to explore a number of clinical relevant questions in the field, in particular whether albumin concentration and duration of dialysis dependency were effect modifiers. To date, two studies i.e. HEMO and the Membrane Permeability Outcomes (MPO) trial contribute the bulk of evidence (>96% weight in a recent Cochrane meta-analysis[44]) about the effects of high flux membranes on outcomes in clinical dialysis. MPO suggested[45] that high flux dialyzers are more efficacious than low flux ones in the subgroup of hypoalbuminemic *incident* patients, i.e. who had been on dialysis for less than three months. On the other hand HEMO did not find a statistically significant effect of high flux dialysis in *prevalent patients*, i.e. those who had been on dialysis for more than 3 months. Independent commentary have attributed the discrepant results to these participant characteristics (lack of focus on a sicker population with low albumin levels, long dialysis dependency) [45–47]. Therefore, we applied flexible modeling techniques to explore the hypothesis that the efficacy of high flux dialysis in HEMO depended on the baseline albumin concentration (treatment by covariate interaction). We also explored the effects of dialysis duration upon this relationship. For these analyses we utilized the patient level data from the study that were provided by the National Institute for Digestive and Kidney Diseases (NIDDK).

The Institutional Review Board (IRB) of the University of New Mexico Health Sciences Center approved the secondary analysis of the flux effect in HEMO (Study ID 13–468 decision of 12/12/2013). All study participants had provided informed consent to participate in HEMO, and the ethics committees/IRBs of participating centers had reviewed and approved the consent form among the other study forms and study protocol. These documents may be downloaded with the HEMO data from the NIDDK repository (https://www.niddkrepository.org/search/study/, study acronym HEMO). Individual HEMO participants were not consented for this secondary analysis, because the data as distributed by the NIDDK has been de-identified and the data use agreement between the investigators of this paper and NIDDK prohibits us from making any contact to identify individuals, families or communities for any purpose (including obtaining an updated consent). The IRB of the University of New Mexico Health Sciences Center waived the requirement for an informed consent for this secondary analysis after reviewing the original consent form that HEMO participants signed upon their enrollment in trial, the data use agreement between the investigators and NIDDK and the associated research protocol submitted to the NIDDK.

## Results

### The Gauss Lobatto is an accurate numerical integration scheme for survival distributions

**Fig 1:**
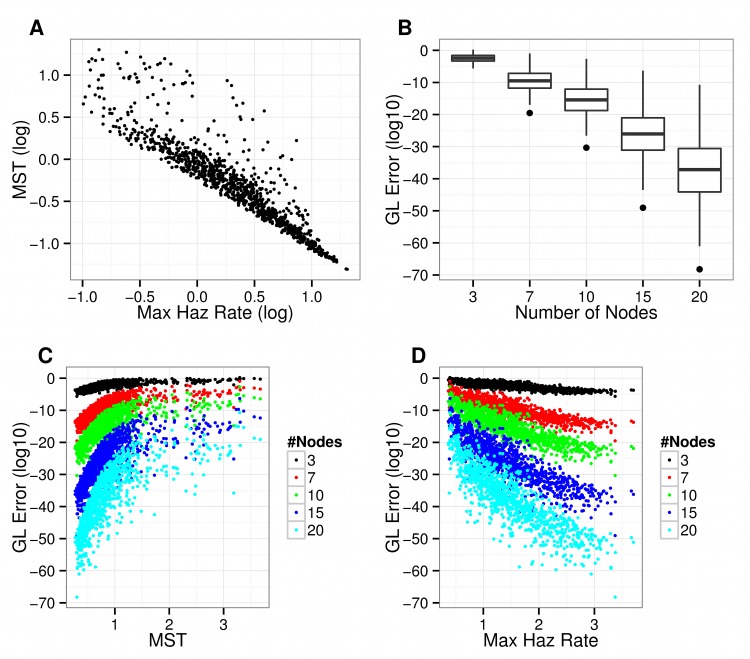
Bounds of the Gauss Lobatto (GL) approximation error for the integration of survival data. A) relationship between (log) MST and the logarithm of the Maximum Hazard rate function for survival distributions with a cubic polynomial log baseline hazard function (B) Box plots of the GL error as a function of the number of nodes in the quadrature rule (C) GL error as a function of the length of the integration interval (taken equal to be equal to the MST for each distribution examined) for different orders of the quadrature rule (D) GL error as a function of the maximum value of the hazard rate for different orders of the quadrature rule.

In our simulations of survival distributions with a log-hazard cubic polynomial function, the MST was inversely related to the maximum hazard rate function as expected (Fig 1A). The expected GL error expressed in (base 10) logarithmic scale was -2.44, -9.51, -15.43, -26.04, -37.18, while the maximum error was 1.03, -0.06, -1.39, -4.51, -8.37, -23.33 for orders of integration of 7,7,10,15,20 respectively. The error declined fairly rapidly with increasing orders of the quadrature rule (Fig 1B). This decline was faster for smaller integration intervals (Fig 1C), but the improvement in performance was not as pronounced for larger integration intervals (e.g. MST> 2 Fig 1C). On the other hand, higher maximum hazard were associated with smaller errors (Fig 1D) as a result of the smaller lifetimes associated with these high hazard ratios (not shown).

### PGAMs generate unbiased and accurate predictions of survival probabilities with acceptable coverage

**Fig 2:**
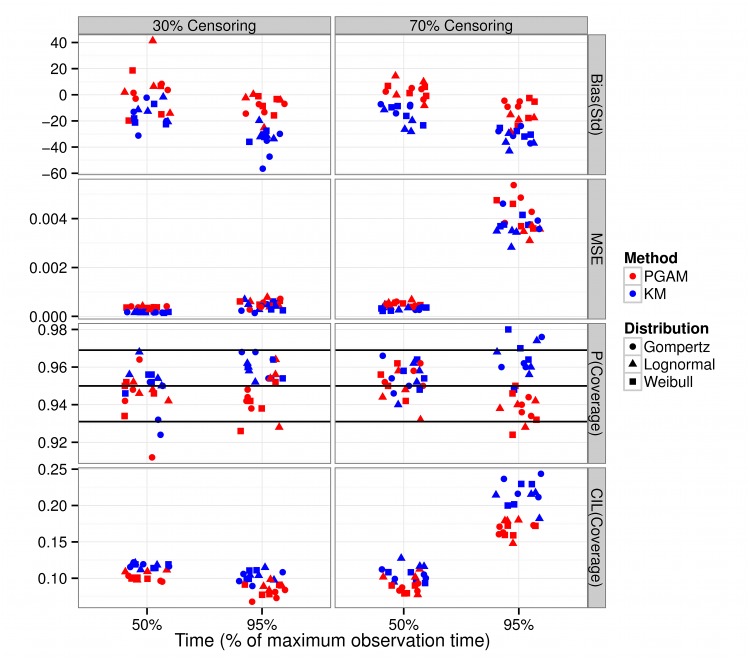
Standardized Bias (top row), Mean Square Error (MSE, second row), p-value coverage (third row) and average confidence interval (CIL) coverage (bottom row) for the survival probabilities from four different baselines of the Gompertz, Weibull and Lognormal distributions. 500 datasets of 300 individuals were simulated for each combination of baseline hazard, parameters and censoring percentage (either 30% or 70%) and were subsequently analyzed with the Kaplan Meier method (blue) and the Poisson GAM (red). The three horizontal black lines in the p-value coverage graph give the nominal coverage (0.95) and ± 2 SE(0.95). Coverage is considered acceptable if the p-values fall within the upper and lower horizontal lines.

Standardized Bias of survival probabilities at the midpoint (50% of the largest observation time) was acceptable (<40%)[26] for both the PGAM and the KM method, irrespective of censoring (Fig 2, top row) and parametric form of the true distribution. At the right tail of the survival curve (95% of the largest observation time), PGAM generated estimates with smaller standardized bias than the KM curve. MSE was similar for both methods (Fig 2, second row) and most of the p-values fell within the acceptable region (Fig 2, third row, upper and lower horizontal black lines). At higher censoring percentages, more KM p-values fell outside the acceptable region compared to the PGAM. Although the CIL was of similar magnitude for both approaches, the PGAM tended to generate shorter intervals (Fig 2, bottom row).

### PGAM based treatment effects are unbiased, accurate and have acceptable coverage

**Fig 3:**
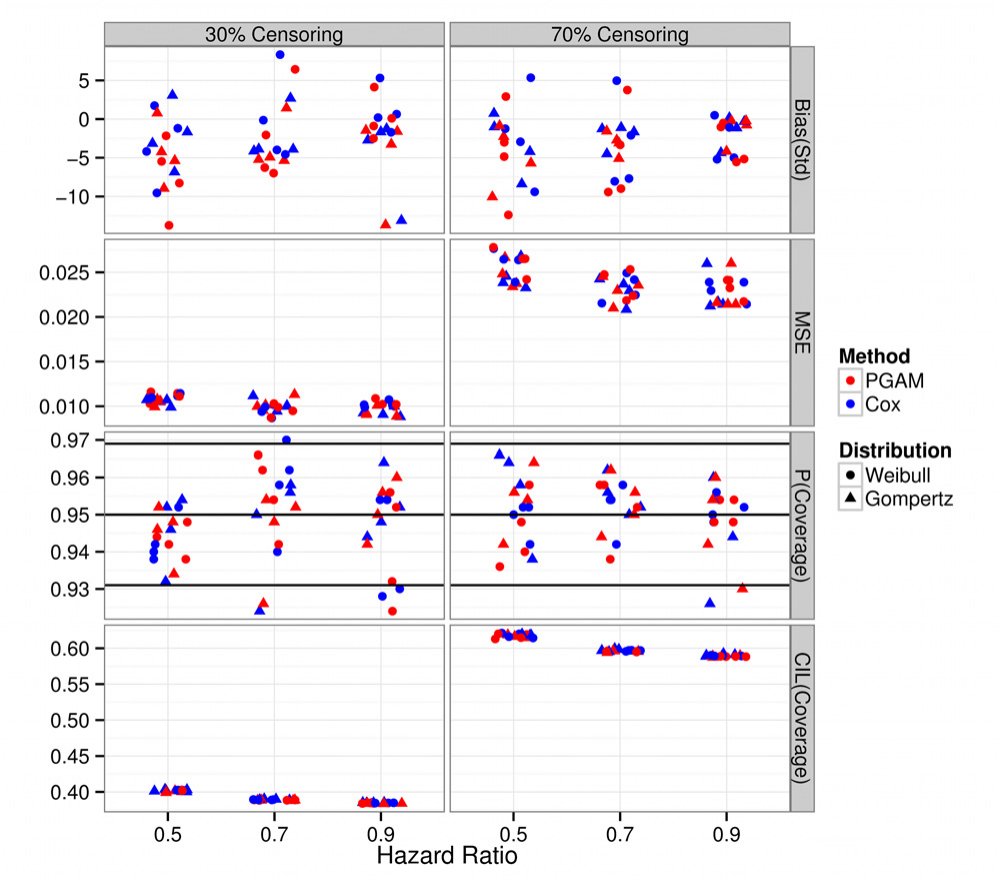
Standardized Bias (top row), Mean Square Error (MSE, second row), p-value coverage (third row) and average confidence interval (CIL) coverage (bottom row) of the Hazard Ratio (HR) from four different baselines of the Gompertz and Weibull. 500 datasets of 300 individuals per arm (total 600 patients) were simulated for each combination of baseline hazard, parameters, HR and censoring percentage (either 30% or 70%). These were subsequently analyzed with the Cox proportional hazards model (Cox, blue) and the Poisson GAM (PGAM, red). The three horizontal black lines in the p-value coverage graph give the nominal coverage (0.95) and ± 2 SE(0.95). Coverage is considered acceptable if the p-values fall within the upper and lower horizontal lines.

HRs were estimated with small standardized Bias by for both PGAM and the Cox model irrespective of censoring (Fig 3, top row), magnitude of the HR and baseline hazard of the true distribution. MSE was similar for both methods (Fig 3, second row) and most of the p-values fell within the acceptable region (Fig 3, third row, upper and lower horizontal black lines). The CIL was of similar magnitude for both approaches and increased by a similar amount when censoring increased from 30% to 70% (Fig 3, bottom row).

**Fig 4:**
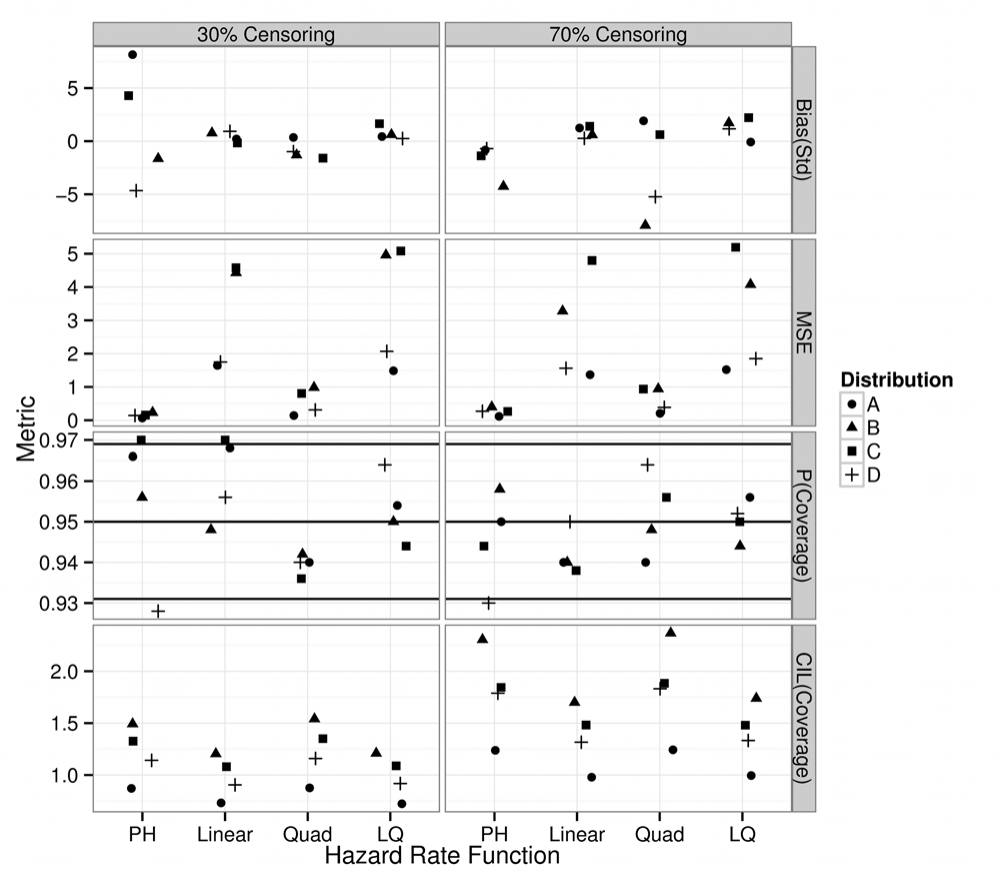
Standardized Bias (top row), Mean Square Error (MSE, second row), p-value coverage (third row) and average confidence interval (CIL) coverage (bottom row) of the Restricted Mean Survival Time (RMST) from four different baselines (A-D) of a general lifetime distribution. 500 datasets of 300 individuals per arm (total 600 patients) were simulated for each combination of baseline hazard, parameters, deviation from the baseline log-hazard (Proportional (PH), Linear, Quad(ratic) and Linear-Quadratic (LQ)) and censoring percentage (either 30% or 70%). These datasets were subsequently analyzed with the Poisson GAM to generate predictions for the Restricted Mean Survival Time (RMST). The three horizontal black lines in the p-value coverage graph give the nominal coverage (0.95) and ± 2 SE(0.95). Coverage is considered acceptable if the p-values fall within the upper and lower horizontal lines.

Predicted RMSTs were unbiased (standardized bias <8%), accurate and with acceptable coverage irrespective of the censoring percentage, baseline, and the form of the deviation from it (Fig 4). In particular the performance of mis-specified PGAMs (PH, Quad) was not quantitatively different from correctly specified PGAMs (Linear, LQ) in terms of coverage.

### Non-proportionality of hazards in the IPASS trial

**Fig 5:**
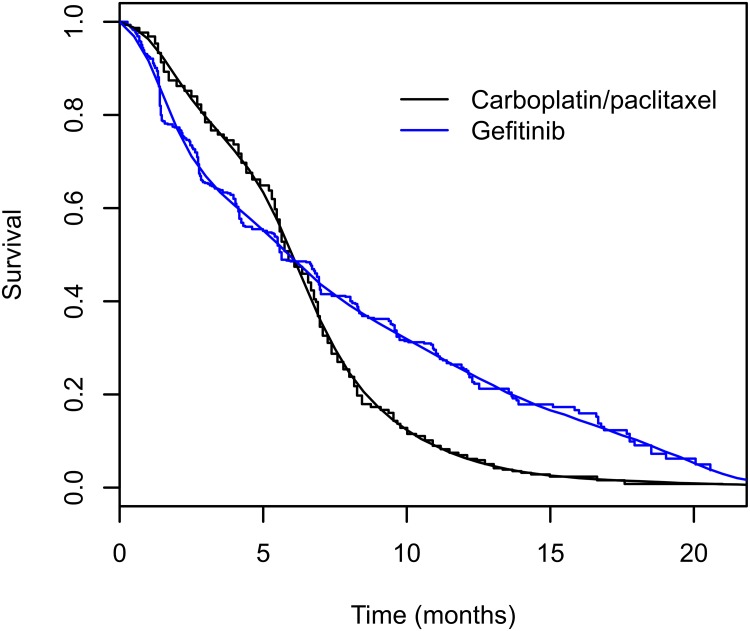
Kaplan Meier curves and superimposed smooth survival curves estimated by Poisson GAM regression in the IPASS trial. Black: carboplatin/paclitaxel arm and Blue: gefitinib arm. The smooth lines are the PGAM survival estimates for the corresponding trial arms. The step functions are the Kaplan Meier curves.

The KM curve and the GAM estimated survival curves in IPASS are shown in Fig 5. There is an apparent violation of the proportionality of hazards assumption as the two KM curves cross. The PGAM estimates of the survival function are smooth curves which closely track the KM curves and cross at the same point as the latter. In the original report of the IPASS trial, the investigators reported a HR of 0.74 (95% Confidence Interval (CI) of 0.65–0.85) in favor of gefitinib[11], which is similar to the Cox model estimate we obtained in the reconstructed data i.e. a HR of 0.73 (95%CI: 0.64–0.83). When applied to the same dataset a proportional hazards PGAM yields nearly identical estimates (Table 1) to the Cox model. Furthermore, these estimates are rather insensitive to the choice of a finer discretization of study time, i.e. splitting time from seven to 20 subintervals did not materially affect the estimates (Table 1).

**Fig 6:**
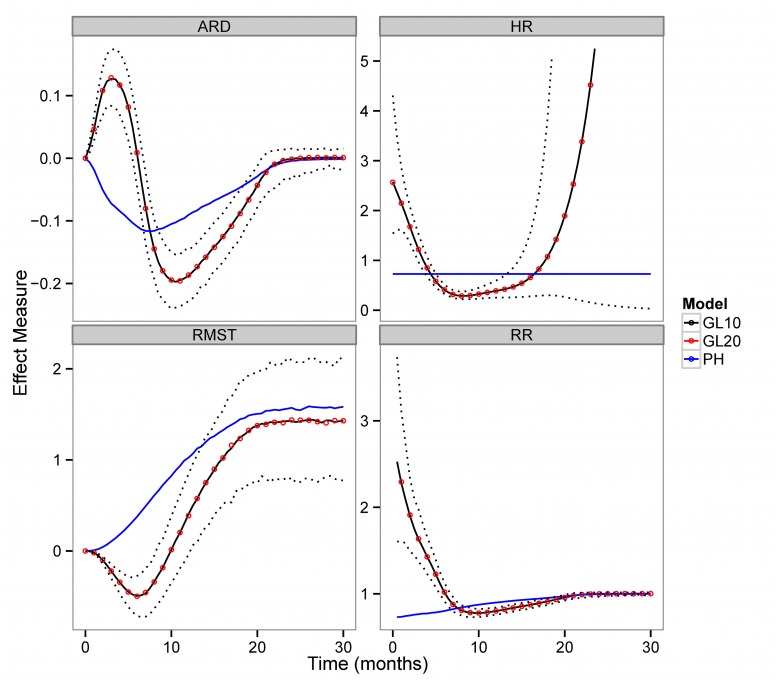
Measures of treatment efficacy in the IPASS trial using proportional hazards (PH) and non proportional hazards PGAMs of two different numerical integration orders: a 10 node Gauss Lobatto (GL10) and a 20 node Gauss Lobatto (GL20) rule. ARD: Absolute Risk Difference, RR: Relative Risk, HR: Hazard Ratio, RMST: Restricted Mean Survival Time. Dotted lines are the associated 95% pointwise confidence interval of the GL10 PGAM.

To address the non-proportionality of hazards in the IPASS dataset we also fit non-proportional hazards PGAMs and contrasted different measures of treatment efficacy (Fig 6) between proportional and non-proportional PGAM models. The crossing of the survival curves is not reflected in the efficacy measures of the proportional hazard fit since the HR is constant (horizontal line in second panel in Fig 6) while the RR slowly increases towards parity with the duration of follow-up. In contrast, estimates from non-proportional PGAM exhibit a bi-phasic decreasing-increasing (HR,RR, RMST) or increasing-decreasing (AR) patterns. Similar to the proportional PGAM case, the non-proportional PGAM estimates are essentially identical when survival time is more finely split. The RMST which measures treatment efficacy on the time scale also differs between proportional and non-proportional PGAM fits. In particular, the early decline in survival time (corresponding to the segment of the KM curves before they cross) is not captured by the proportional hazards model. RMSTs do not change after the 20th month since the overwhelming majority of patients in both arms have experienced the event of interest. The proportional model yields more optimistic estimates of the gain in survival time than the non-proportional model in IPASS. Approximating the MST with the RMST at 40 months we obtained estimates of 1.60 months (95%CI 1.01–2.24) v.s. 1.42 (95% CI: 0.73–2.12) respectively. Though the magnitude of the difference is not clinically meaningful, it may have important implications for Health Technology Assessments (HTA). In the latter, the incremental survival benefit relative to incremental cost is evaluated against cost effectiveness thresholds (e.g. between 50–100000 dollars per QALY in the US[48,49]) before recommending the use of a new healthcare technology. Depending on the difference in costs one can envision scenarios in which methodology dependent differences in the calculated values of the MST impact policy decisions for costly technologies[50,51].

### Center effects, multiple time scales and subgroup analyses in the HEMO trial

**Fig 7:**
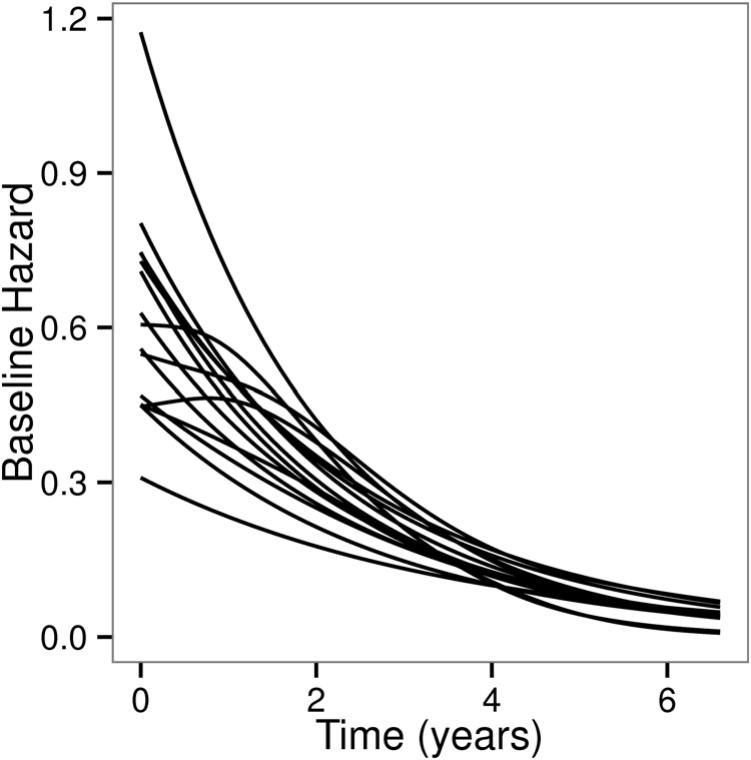
Baseline hazard functions in the 15 centers of the HEMO trial produced by a 10 node Gauss Lobatto quadrature rule.

PGAM estimates of HRs adjusted for baseline covariates in HEMO were insensitive to the granularity used to split survival time. These estimates were numerically similar to those obtained with the stratified, Cox model (Table 2) used in the primary analysis of HEMO. In particular, the treatment effects of the two interventions (dose and membrane flux) estimated by the PGAM were identical to the ones obtained with the Cox model. Estimates of the baseline hazard function stratified by center are shown in Fig 7. With the exception of three centers, these appear to decay exponentially with time, though both the intercept and the rate of decline differ among centers implying the existence of moderate to large center effects. However, a random effects analysis to look for flux by center interactions in the HR did not show evidence for variability of the efficacy of the flux intervention by center (not shown).

**Table 2:** Estimates of the Hazard Ratio and associated 95% CI in the HEMO trial.

	Cox		GL7		GL10		GL20	
Variable	HR	95% CI	HR	95% CI	HR	95%CI	HR	95%CI
High Kt/V	0.96	0.84–1.10	0.96	0.84–1.09	0.96	0.84–1.09	0.96	0.84–1.09
High Flux	0.92	0.81–1.05	0.92	0.81–1.06	0.92	0.81–1.06	0.92	0.81–1.06
Age (per 10)	1.41	1.33–1.50	1.42	1.33–1.51	1.42	1.33–1.51	1.42	1.33–1.51
Female	0.85	0.73–0.98	0.85	0.73–0.98	0.85	0.73–0.98	0.85	0.73–0.98
Black	0.77	0.66–0.91	0.77	0.66–0.91	0.77	0.66–0.91	0.77	0.66–0.91
Diabetic	1.29	1.11–1.50	1.29	1.11–1.5	1.29	1.11–1.5	1.29	1.11–1.5
Duration	1.04	1.02–1.06	1.04	1.02–1.05	1.04	1.02–1.05	1.04	1.02–1.05
ICED	1.37	1.25–1.50	1.38	1.27–1.51	1.38	1.27–1.51	1.38	1.27–1.51
Alb (per 0.5 g/dl)	0.51	0.43–0.62	0.53	0.45–0.64	0.53	0.45–0.64	0.53	0.45–0.64
Alb X Time interaction	1.11	1.04–1.19	1.09	1.02–1.16	1.09	1.02–1.16	1.09	1.02–1.16

Cox: Cox proportional hazards model analysis, GL7-GL20: estimates produced by a Poisson GAM using 7, 10 or 20 nodes for the numerical integration of the cumulative hazard function. ICED: Index of Coexistent Disease, Alb: albumin. Analyses were stratified by study center, which implies that the Cox model used a separate baseline hazard for each participating center, while GAM models incorporated explicit interactions between study center and observation time. Variables used to adjust estimates were prespecified in the HEMO analysis protocol.

**Fig 8:**
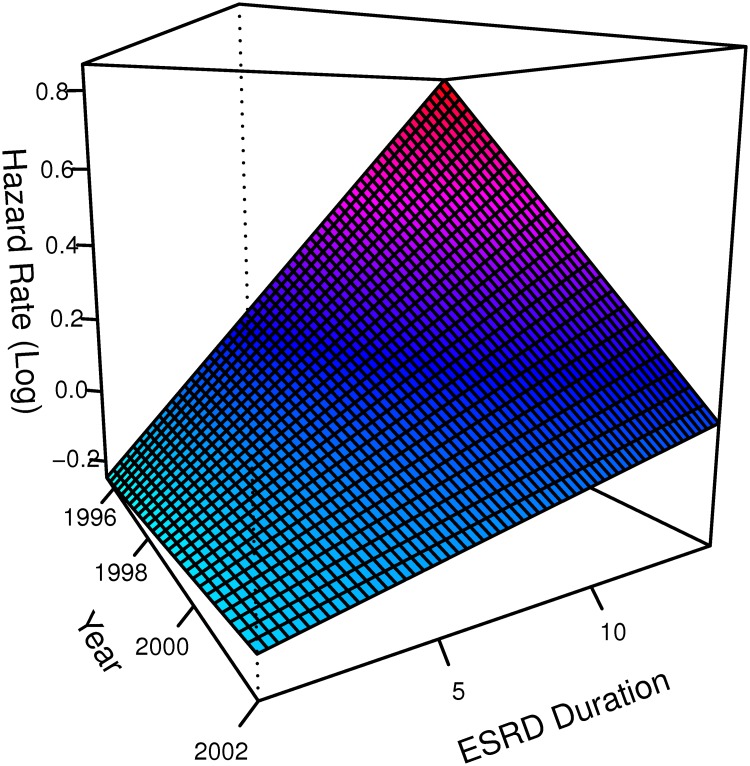
Log hazard rate function for the interaction between calendar time and duration of ESRD at the beginning of HEMO. This was estimated via a tensor based smooth in a stratified by center PGAM adjusting for all prespecified baseline covariates in HEMO, a general (not linear) interaction between baseline albumin concentration and observation time, the combined effects of albumin and disease duration (as a tensor product smooth) and the modification of the latter by high flux dialysis.

To model the effects of the duration and calendar time scales we used a two-dimensional tensor product of smooths[10]. With such a construction, models used to analyze HEMO are adjusted for secular trends in mortality of dialysis patients (calendar time scale), dialysis dependency duration (duration time scale) and their interaction (as secular rates may have differentially improved for patients with different ESRD durations). The interaction between in calendar time and disease duration is shown in Fig 8 allowing for several observations. First, within each year the hazard rate is higher for patients who had been on dialysis longer. At the same time, mortality v.s. dialysis duration is generally lower for patients dialyzing in later years, i.e. outcomes improved for dialysis patients throughout the 90s. Finally, improvements are larger for patients who had been on dialysis for longer periods of time e.g. contrast the steepness of the decline in the log hazard ratio for patients who have been on dialysis for 10 years compared to those who had been dialysis dependent for five years or less.

**Fig 9:**
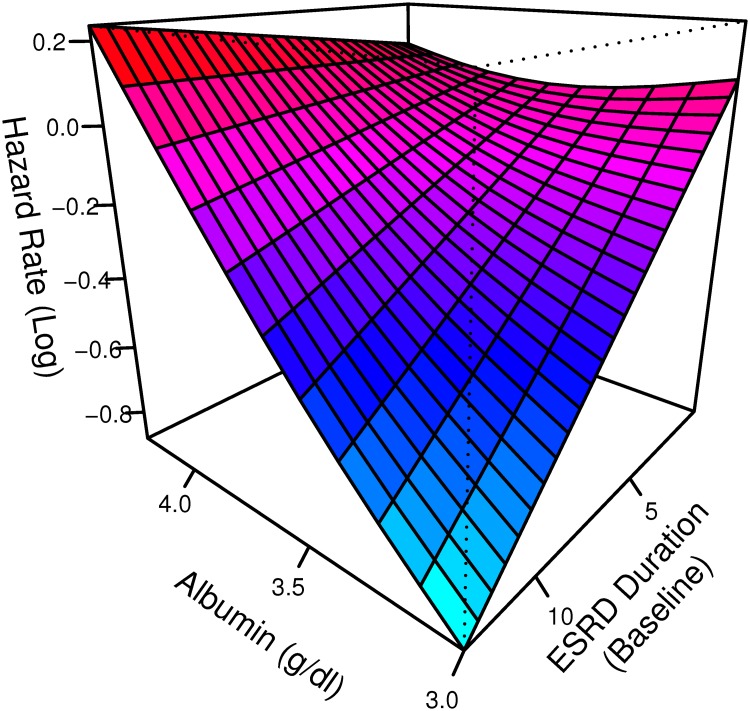
Log hazard rate function for the interaction between albumin and duration of ESRD at the beginning of HEMO and flux. This was estimated via a tensor based smooth in a stratified by center PGAM adjusting for all prespecified baseline covariates in HEMO, a general (not linear) smooth interaction between baseline albumin concentration and observation time, the interaction between albumin and disease duration (as a tensor smooth) and the triple interaction between albumin, disease duration and high flux arm assignment (shown in the Fig). A 10 node Gauss Lobatto quadrature rule was used to numerically integrate the cumulative hazard function.

To examine the modification of the effects of HF membranes by dialysis duration and baseline albumin concentration we included tensor product terms for albumin, duration of dialysis and the interaction between this tensor term and high flux assignment. Both terms were statistically significant (p<0.001 and p = 0.049 respectively); a visual representation of the effect modification of flux by albumin levels and dialysis dependency is depicted in Fig 9. This response surface shows that HF dialysis was associated with numerically smaller log hazard ratios, indicative of a more beneficial effect, in patients who had been on dialysis longer and those with lower albumin concentrations. However these relationships are not constant across the entire range of these covariates; whereas patients with low albumin (less than 3.5) exhibit a rather steep relationship between the log-hazard rate ratio and dialysis duration, the steepness decreases for higher albumin values, and may even change direction when the latter is greater than 4 g/dl. To aid visualization of the complex relation between flux, albumin and dialysis duration we created continuous loop animations for the adjusted hazard rate function across the range of albumin and dialysis duration. These are shown both in the log (S1 Fig) and linear scales (S2 Fig). Where the high flux albumin-duration surface (red) is below the low flux surface (almost invariably when albumin < 3.5 g/dl), the hazard ratio will be below one (suggesting a protective effect of high flux dialysis) and vice versa. Hence, high flux dialysis is associated with a beneficial effect (smaller hazard rate) on survival in hypo-albuminemic dialysis patients, but a neutral effect in those with normal albumin.

**Fig 10:**
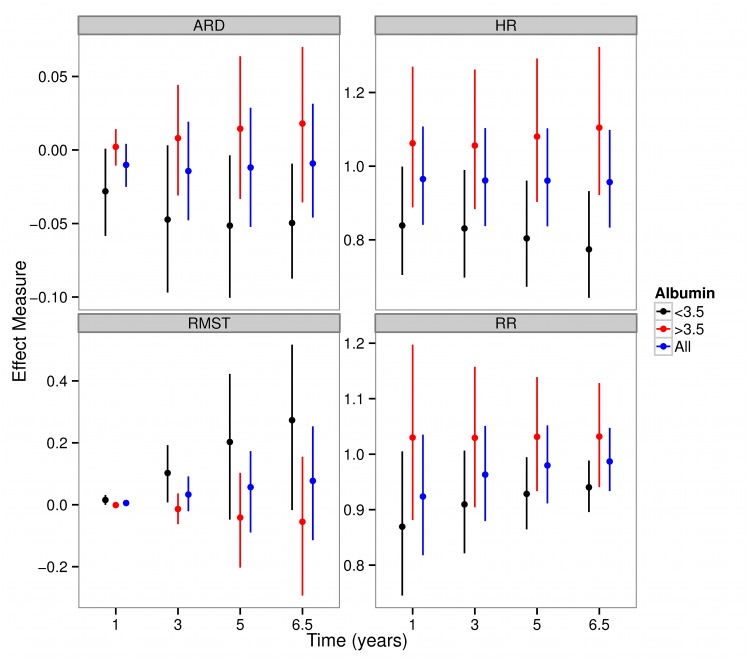
Adjusted measures of treatment efficacy in the HEMO trial using the corrected group prognosis method in the subgroups patients with albumin less than 3.5 g/dl, greater than 3.5 g/dl and the entire HEMO sample. A 10 node Gauss Lobatto quadrature rule was used to numerically integrate the cumulative hazard function. ARD: Absolute Risk Difference, RR: Relative Risk, HR: Hazard Ratio, RMST: Restricted Mean Survival Time. Dotted lines are the associated 95% pointwise confidence interval of the GL10 PGAM.

In order to quantify the divergent effects of dialysis in hypo-albuminemic patients we computed *population averaged* measures of treatment efficacy in the subgroups of patients with albumin ≤ 3.5 v.s. > 3.5 g/dl and the entire HEMO sample. High flux dialysis is approximately associated with a 5% absolute risk reduction, a hazard ration of 0.77, a relative risk of 0.94 and a RMST of 0.27 years after being exposed to HF dialysis for 6.5 years (the end of follow up in HEMO). In contrast, the effects on patients with higher albumin concentrations and the overall HEMO cohort are smaller (Fig 10). The extended PGAM model used to derive Figs 9 and 10 incorporated six proportional covariates, 15 stratification terms, a flexible interaction of albumin with time, three tensor product terms modeling the impact of the calendar and duration time scales as well as the effect of albumin and dialysis duration and its modification by flux. In spite of the large number of terms, the penalized GAM procedure resulted in a model with 57.48 estimated degrees of freedom corresponding to 15.15 events per degree of freedom as there were 871 events in HEMO.

## Discussion

We have presented an overview of the PGAM approach for flexible modeling of survival data using standard software. Theoretical considerations, numerical explorations and simulations demonstrate that the PGAM yields unbiased and accurate estimates of survival probabilities, hazard ratios and alternative treatment effects. We have shown how multiple measures of treatment efficacy can be obtained using this approach in both adjusted and un-adjusted analyses in real world datasets. PGAMs support subgroup analyses, center effects and multiple time scales while accounting for non-proportional hazards. By augmenting the hazard ratios conventionally reported by trialists, PGAMs have the potential to support the inferential goals of multiple stakeholders involved in the evaluation and appraisal of clinical trial results[52].

### The relation of the PGAM to the Cox Model and the Kaplan Meier procedure

In spite of their different theoretical underpinnings PGAMs yield nearly identical estimates to the KM curve and HRs obtained by Cox proportional hazard models in simulations and clinical trial datasets. This is hardly surprising because, both these classical survival analysis methods are numerically equivalent to Poisson regression[16,53–57], if the observation time is split at the unique failure times, the baseline hazard is assumed to be piecewise constant and the trapezoidal rule is used to numerically integrate the hazard function. From a numerical perspective the PGAM approach we utilized differs from the KM and the Cox model only in these technical but crucial aspects. In particular, we split time at the nodes of the GL quadrature rule used to numerically integrate the hazard function, while employing a penalized smooth spline basis, rather than step functions, to model the log-hazard rate. These technical innovations allowed us to substantially reduce the computational resources used to fit these models, addressing a major limitation of Poisson regression for survival data[9,16]. Our theoretical and numerical explorations are the first to demonstrate the utility of the GL to facilitate PGAM analyses of survival data. Penalized fitting offers a parsimonious option for realistic modeling of survival data without spending a large number of degrees of freedom during model fitting. The latter aspect is important when one is considering multiply adjusted models, subgroup analyses and multiple time scales which all require degrees of freedom to be spent for their estimation.

### PGAMs v.s. other flexible approached for modeling survival

In the context of flexible, yet parametric alternatives to the Cox model, the major alternatives to the PGAM are shown in Table 3. The method most closely related to the PGAM is the “stgenreg” [18]. While this manuscript was under review, these authors provided additional details about their methodology[58] allowing a more complete comparison. Both methods use Gaussian quadrature to integrate the baseline hazard, i.e. Legendre[58] v.s. Lobatto (PGAM) but differ in the estimation method (REML in PGAM but Maximum Likelihood (ML) in “stgenreg”). The PGAM but not “stgenreg”, allows modeling of correlated outcomes via frailty terms. Both methods are in principle able to support multiple measures of treatment effect either directly (during model estimation) or post-estimation by simulation but the PGAM’s repertoire is wider and includes integrated measures such as the RMST. Direct, flexible modeling of the Cumulative Hazard function via maximum likelihood is also possible (“stpm2”[59]) with HR and RMST measures derived post-estimation via numerical differentiation and integration respectively. There exist Bayesian alternatives to the PGAM framework (BayesX[60]) to the PGAM framework. BayesX builds on the generalized mixed model approach to penalized fitting in order to carry out survival analyses of either grouped or continuous time survival data. The former are fit using logistic regression (an approach well established in the literature[61]), which is also accessible from the GAM framework by substituting the Poisson with logistic regression. We did not pursue this approach in our work. When smooth baseline hazards are assumed, BayesX directly optimizes a numerically integrated likelihood, hence it is most properly viewed as a Bayesian extension of the “stgenreg”[18] approach. A major shortcoming of BayesX is that it uses the trapezoidal rule over a uniform grid to numerically integrate the baseline hazard. This leads to large datasets and slow fitting times even when REML estimation (rather than Markov Chain Monte Carlo) is used. Out of all the possible alternatives considered (Table 3) it is only the PGAM that has specialized implementations for “big data” and the only one that offers extensive opportunities for parallelization using a variety of architectures (multi-core processors, or clusters) when fitting the model. Furthermore the post-estimation simulations are “embarrassingly” parallelizable[62,63] using a variety of hardware architectures opening up the opportunity to derive personalized treatment effect predictions after multivariable adjustment. Considering the features of all the “competing” approaches in Table 3, it seems that the PGAM offers the best combination of analytic capabilities and computational advantages.

**Table 3:** Options for parametric, flexible modeling of survival data.

	GAM (this paper)	*stgenreg*[18,58]	*BayesX*[60]	stpm2[59]
*Default Scale*	Log-hazard	Log-hazard	Log-hazard	Log-Cumulative Hazard
*Time*	Continuous (Poisson)Grouped(Logistic) ‡	Continuous	Continuous (Poisson)Grouped(Logistic)	Continuous Time
*Delayed Entry*	Yes	Yes	Yes	Yes
*Non-proportional hazards*	Yes	Yes	Yes	Yes
*Measures of (treatment) effect*	1. HR (estimated) 2. RMST, ARR, RR, R (prediction by simulation post- estimation)	1.HR, R (estimated)	1. HR (estimated) 2. RMST, ARR, RR, R (prediction by simulation post—estimation) ‡	1. CHR, R (estimated) 2. HR,RMST (post-estimation)
*Excess mortality models*	Yes 1. During model fitting, by fitting the identity rather than the log link for PGAMs‡ 2. Prediction of excess (relative) Mortality by simulations after model fitting	Yes Specified when fitting the model	Yes Predictions of excess (relative) mortality by simulation after model fitting‡	Yes Specified when fitting the model
*Flexible function basis for modeling the (baseline) log-hazard*	Penalized, Cubic or Thin Plate Splines or user defined basis	Restricted Cubic Splines	Piecewise Exponential or P-splines	Restricted Cubic Splines (Log-cumulative hazard)
*Ability to handle multiple time scales simultaneously*	Yes	Yes‡	Yes‡	Unclear
*Complex multidimensional interactions between continuous and discrete covariates*	Yes	No	Yes	No
*Random Effects*	Yes	No	Yes	No
*Correlated Outcomes*	Yes 1. Frailty (adjusted effects) 2. Population averaged effects by simulation post-estimation	Yes 1. Robust (cluster) Standard Errors 2. Frailty not currently implemented	Yes 1. Frailty (adjusted effects) 2. Population averaged effects by simulation post-estimation‡	No
*Estimation*	REML for GAMs GCV for GAMs‡	ML (direct optimization)	REML/MCMC	ML(direct optimization)
*Numerical quadrature*	Gauss Lobatto	Gauss Legendre	Trapezoid Rule	Not Applicable
*Availability of specialized “Big Data” implementations*	Yes	Unclear	No	Unclear
*Scalability and opportunities for parallelization*	Yes 1. Parallel threads on shared memory multi-core machines (estimation) 2. Clusters (OpenMP[99]) for “Big Data” applications (estimation) 3. “Embarassingly” Parallel[62,63] simulations post-estimation executed in multi-core machines, clusters or even GPGPUs	Unclear	Partial Parallel chains when using MCMC simulation for Bayesian model fitting	Unclear

^‡^ Not pursued by the authors

*Abbreviations*: ARR (Absolute Risk Reduction), CHR (Cumulative Hazard Ratio), GAM (Generalized Additive Model), GCV (Generalized Cross Validation), GPGPU (General Purpose Graphics Processing Unit) HR (Hazard Ratio), MCMC (Markov Chain Monte Carlo), ML (Maximum Likelihood), OpenMP (Open Multi-Processing), PGAM(Poisson Generalized Additive Model), R(Relative Survival), REML (Restricted Maximum Likelihood), RR (Relative Risk).

### PGAMs, alternative measures of treatment effect and the multistakeholder environment of RCTs

An important advantage of PGAMs is the capability to calculate all conceivable alternatives to the HR through simulation, which in our view this provides a major justification for their use in RCTs. The predominance of the HR in the clinical literature overshadows the deficiencies complicating its use as previously pointed by authors from biostatistical[7,64] and clinical perspectives[4,65]. The need for multiple measures is underscored by the observation that HRs may be large even when the actual benefit in survival time is small. This was evident in our analysis of IPASS in which a HR of <0.80 translated in only small improvements in measures of (restricted) mean survival time. However the major criticism in the current era involves the indirect manner in which HRs support the inferential goals of non-physician stakeholders involved in the evaluation and appraisal of clinical trial results. For these stakeholders (third party payers, policy makers and patients), the effect of treatment on survival probabilities or even the survival time are more relevant. Before these alternative measures of treatment efficacy can be reported however, the baseline hazard should be estimated along with a relative measure of treatment effect (e.g. the hazard ratio). Hence, even if the proportionality of hazards assumption is verified, the PGAM approach, that estimates the same numerical hazard ratio as the Cox model, has a distinct advantage in the current environment in which clinical trials are undertaken. In particular, *current trends in best medical practices*[5,6,66–68] *and legal provisions in the US*[69] *put increasing emphasis on sharing clinical trial information with patients*. However there is still a knowledge gap concerning the appropriate measure to communicate to patients[70–72]. The PGAM addresses this gap by providing a method that can analyze clinical trial data in a rigorous fashion while generating a variety of effect measures to facilitate patient communication and clinical decision making.

### PGAMs and the proportionality of hazards assumption in survival analysis

In the case of non-proportional hazards some investigators have argued that the overall HR computed by a Cox model be given an average (over the observed death times) interpretation and have even proposed estimation procedures based on time weighting schemes[73]. This view may appeal to audiences accustomed to the use of the HR, yet it is not is not particularly satisfactory[64]. In particular, the average HR is a function of the follow-up time leading to the counterintuitive situation in which increasing the duration of follow-up may lead to estimates of the HR that are furthest from the truth[74]. We have also illustrated this shortcoming when discussing[75] the analysis of a real world dataset in which the violation of the proportionality assumption[76] may critically impact the selection of treatment modalities by both clinicians and patients. In such a situation, fitting a non-proportional hazard model is the mathematically correct procedure, even though by being a function of time this function will no longer provide a single numerical summary of the treatment effect in the trial. Nevertheless a PGAM one can generate the curve relating the HR to time, providing further insights into the effects of the interventions studied[75]. Such curves may be of great utility when communicating the temporal risk tradeoffs of a given intervention (e.g. kidney transplantation[77] or the choice between alternative forms of maintenance dialysis[78]) and have traditionally been derived with piecewise exponential models. The PGAM is an extension of this approach which has the advantage of providing visually smooth reconstructions of the HR curve without making arbitrary choices about the point in time in which the HR may change magnitude or direction.

Our simulations suggest that the PGAMs are robust with respect to mis-specification of a non-proportional hazards model in the case of proportional hazards as long as an integrated measure of treatment efficacy is used for results reporting. Alternatively one fit a non-proportional PGAM, and test the coefficients of its time-varying part for proportionality as we comment in the Methods. This would amount to a simultaneous testing of the proportionality assumption and a test of significance for a non-null treatment effect, an idea that was first proposed more than 20 years in the context of spline estimation for survival data[79,80]. Nevertheless it may be more appealing to use shrinkage smoothers[10] which can automatically select a proportional model if the source data do not suggest a variation of the log-hazard with time. This is rather straightforward, technical application of the PGAM that can be explored in contexts in which the RSMT is a less desirable treatment effect summary than the HR.

### PGAMs for adjusted or subgroup analyses in RCTs

Though multivariable adjustment and subgroup analyses are not overwhelmingly endorsed by trialists or regulators[81], there are widely applied in clinical trials[82–84]. Our adjusted and subgroup analyses in the HEMO trial highlight several features of PGAMs in this context, including the robustness of the estimates to the granularity of time-splitting, their ability to visualize the baseline hazard and center effects in stratified models and the support of very general, even multidimensional, interaction terms between treatment and baseline covariates. The latter allows one to introduce interactions without making any assumptions about the latter’s direction, magnitude or functional form. In our opinion these represent significant advantages for the defense of the “objectivity” of adjusted/subgroup analyses with PGAMs. As previously pointed out[21,85], the tension implicit in these analyses involves a compromise between the conflicting goals of detailed modeling that makes the most out of the available data, while at the same time guarding against “data fishing”. We demonstrate the ability of PGAMs to achieve such a balance in the subgroup analysis of the effects of membrane flux on outcomes of hypoalbuminemic patients in HEMO. Our finding of a benefit of high flux dialysis on patients with serum albumin of 3.5 gm/dl is in remarkable agreement with the findings of the MPO controlled trial[45], once the differences in the albumin assays used in these two trials are accounted for[86,87]. However this association emerged from visualization of general continuous interaction between baseline albumin and serum flux instead of specifying a cutoff for the categorization of albumin based on the MPO results.

In our analyses the direct adjusted HR obtained by averaging over the covariate distribution of the HEMO study participants is nearly identical to an unconditional PGAM estimate. The explanation for this observation may be found in the seminal paper by Gail et al[88] which considered the bias in the estimation of treatment effects when covariates were omitted from non-linear regressions of randomized experiments. In particular, unconditional estimates of the mean parameter in a Poisson regression (e.g. the log-hazard in a PGAM) are unbiased against their covariate averaged counterparts for large samples. This general feature of Poisson and thus PGAM regression suggests they may be able to fill an important gap between unconditional, unadjusted effects for policy making and adjusted ones that inform care decisions for specific patient subpopulations. Specifically, we suggest the use of adjusted PGAM analyses to account for imbalances in prognostically important covariates and to explore treatment effect heterogeneity in subgroups so as to get “as close as possible to the clinically most relevant subject-specific measure of treatment effect”[89]. Subsequently one could average these adjusted estimates to generate overall treatment effects which are the focus of clinical trialists and regulators. We use the HEMO trial to illustrate this PGAM based approach in generating the complementary treatment effect measures for these dissimilar audiences. In HEMO averaging over the model incorporating albumin by flux interaction yields an overall treatment effect which does not deviate from the unconditional estimate, and thus does not alter the interpretation of the trial results. At the same time the adjusted estimate provides an insight into the treatment effects on a particularly susceptible, medically important subpopulation with low serum albumin levels. Since HEMO and MPO are the largest trials to date to examine the effects of high flux dialysis on outcomes[44], this subgroup analysis of HEMO may be relevant for strengthening the evidence basis of clinical guidelines[90–92] which support the use of high flux dialyzers in patients with low albumin.

### PGAMs, multiple time scales and secular trends in RCTs

Randomized controlled trials are seldom adjusted for secular trends or drift during the duration of the study because the presence of a control (comparison) arm is thought to guard against biases introduced by drift. Nevertheless, secular trends may affect the internal validity i.e. by affecting (unmeasured) confounders and risk modifiers that limit the effects of treatment[93]. More importantly, secular trends of improvement of the outcomes in the comparison group may decrease the trial’s sensitivity to detect a true effect[94], or even tip the risk benefit balance so that an once efficacious treatment is associated with relatively worse outcomes. Though secular trends did not affect the treatment effect estimate in HEMO, we did observe large secular trends in the risk of death during the course of the study. Analysis from the United States Renal Data System (USRDS) registry have shown that mortality of dialysis patients declined during the 90s (the period during which HEMO was being carried out)[43], while these secular reductions in mortality were numerically higher in patients with longer dialysis dependency USRDS (e.g. contrast Fig.s5.1 and 5.4[43]) a pattern that was seen in our analysis of the HEMO trial. As randomized controlled trials cannot be completely insulated against secular trends, the ability of PGAMs to incorporate multiple scales may make them particularly useful for the analysis of designs that are particularly susceptible to drift(e.g. long duration or community level trials[93,95,96]). Nevertheless, secular trends may also be seen in individually randomized RCTs e.g. in depression[97] and cardiovascular disease[94,98] so that the ability of the PGAM to account for multiple scales should be strongly contemplated, at least for the secondary analyses of such trials.

### Limitations and future extensions

Our focus in this work is more applied than theoretical so we mainly demonstrated how PGAMs can be applied to address practical problems of the analyst facing actual trial data. However, we did not pursue mathematically rigorous derivations that address technical issues including the optimality of the Gauss Lobatto quadrature rule and the degree of time splitting to apply for a *particular dataset*. Though these may seem major limitations, their implications for applications are likely limited. In particular, the Gauss quadrature formulas are among the most computationally efficient ones, so it is unlikely that an alternative choice would make a substantial difference on results. Furthermore, one may always split time using a finer grid if the accuracy of the PGAM results is in question. Theoretical considerations and our numerical exploration of the GL error suggest that for most realistic datasets discretizing observation time to 10–20 intervals should give an adequate approximation. Future research that considers the numerical accuracy and error estimates of quadrature schemes should be pursued to elucidate such issues. Until the theoretical resolution of these issues, the robustness of PGAM estimates should be subjected to a sensitivity analysis as with all numerical methods.

## Conclusions

In this paper we illustrate the use of the Poisson Generalized Additive Model approach for flexible modeling of survival data using standard software. We have shown how multiple measures of treatment efficacy can be obtained from a single pass through the data in both adjusted and un-adjusted analyses. Our analyses highlight the flexibility of PGAMS in supporting subgroup analyses, incorporating multiple time scales while accounting for non-proportional hazards. By augmenting the hazard ratios conventionally reported by clinical trialists, PGAMs have the potential to support the inferential goals of multiple stakeholders involved in the evaluation and appraisal of clinical trial results.

## Supporting Information

S1 Fig Continuous loop animation of the adjusted log hazard rate function of patients assigned to high v.s. low flux dialysis as a function of baseline albumin concentration and dialysis dependency duration upon entry into the study. (MP4)

S2 Fig Continuous loop animation of the adjusted hazard rate function of patients assigned to high v.s. low flux dialysis as a function of baseline albumin concentration and dialysis dependency duration upon entry into the study. (MP4)

S1 Dataset Reconstructed Individual Patient Data in the Gefitinib arm of the IPASS trial. (TXT)

S2 Dataset Reconstructed Individual Patient Data in the Carboplatin arm of the IPASS trial. (TXT)

S1 Text R script to reproduce the analysis of the IPASS trial presented in the text. (TXT)

S1 Appendix (DOCX)
