# Supplementary material for: Analysis of Time to Event Outcomes in Randomized Controlled Trials by Generalized Additive Models
Source: PLoS One. 2015 Apr 23;10(4):e0123784. doi: 10.1371/journal.pone.0123784 (PMC4408032; doi:10.1371/journal.pone.0123784)
Supplement: S1 Appendix — (DOCX) [file pone.0123784.s006.docx]

# S1 Appendix

## A1. Literature Survey

The close connection between survival analysis via proportional hazard modeling and Generalized Linear Models (GLMs) was made very early after the publication of the corresponding landmark papers [1,2]. Since then, the connection has been re-discovered a number of times:

- Breslow[3] considers the proportional hazard model as a discrete time logistic regression in which discrete probability masses are put on the (ordered) set of observed failure times. The use of the logistic regression was subsequently reported by others[4–6] during the 70s.
- Breslow[3,7] discretized a continuous time model by forcing the hazard to be constant between successive failure times. This piecewise exponential (PE) model was later recognized to yield estimates identical to Poisson regression for rate/exposure data[8].
- Holford[9,10] introduced Poisson regression models (PR) for survival analysis and proved the equality of Maximum Likelihood Estimates (MLE) from the PR, the PE and the Maximized (profile) likelihood of the PH model.
- It appears that the connection between survival analysis, lifetable methods and GLMs (Poisson and Logistic) has been utilized to fit the PH model prior to the development of PH specific software[11–13]. At least one early landmark RCT in the author’s field appears to have been analyzed with the PH using GLM-like software[14]. Clayton[15] presents one of the most complete “theory to implementation” treatments of the GLM-survival connection with software available in 1983. This paper is the first to highlight the flexibility of the approach and a number of possible extensions but also pointed out the computational problems for large (for the computers available in the early 1980s) datasets.
- A thorough and rather general treatment of the relationship between survival analysis, the PHM, logistic and Poisson regression was given by Efron[16] who also reviewed the relevant literature up to that time. In this paper, commonalities among the logistic and Poisson GLM approaches to survival analysis were highlighted: the explicit discretization/partitioning of the time axis to disjoint interval and the introduction of specific parameterization of the underlying hazard function (via step or low order polynomial functions).

In the backdrop of this extensive early literature, Carstensen[17] made a forceful argument for the near irrelevance of the PH formulation in the era of modern computers and argued for the adoption of the Poisson formulation on the basis of simplicity and insightfulness of presentation. These advantages had also been noted by Efron almost 20 years earlier[16]. Carstensen also explored suitable adaptations for the (simultaneous) analysis of multiple temporal scales, stratified models, non-proportional hazards, time-dependent covariates and even multi-state models by combining the use of regression splines in the Poisson GLM framework with ideas borrowed from the field of demography. Other investigators have advanced the GLM approach by using smoothing splines to estimate the hazard function from a Bayesian perspective. The common unifying theme of this literature (an overview appears in Section 3.19 of the relevant survey[18]) is the exploitation of the relationship between random effects models and smoothing splines in order to estimate the degree of freedom of the log-hazard function in a data-driven fashion. This is in contrast to the conventional GLM approach[15–17] in which the degrees of freedom of the hazard function is fixed beforehand. We note that a Bayesian, much slower, adaptation of GAMs for the modeling of survival has also been described in the literature [19,20] and released both as a standalone package (*BayesX*[21]) and as an *R* frontend. In the aforementioned work, the considerations and the implications underlying the choice of the numerical integration scheme (usually the trapezoidal rule) used to approximate the cumulative hazard function are not considered.

## A2. Measures of treatment effect in survival studies

In a comparative evaluation setting, one would want to quantify how dissimilar the survival is among two treatment groups. Depending on the modeling framework adopted and the scale (absolute or relative) used, the following efficacy measures may be calculated for two hypothetical treatments A v.s. B:

- Relative Risk (RR) at time *t* i.e. the ratio of patients who have experienced an event among those receiving A v.s. B: $RR(t)=\frac{1-S_{A}(t)}{1-S_{B}(t)}$
- The Relative Survival i.e. the ratio of survival in patients receiving A v.s. B: $R(t)=\frac{S_{A}(t)}{S_{B}(t)}$ If the survival of the second group is the expected survival among a disease free population, then this ratio corresponds to a relative (excess) mortality model.
- The Absolute Risk difference (ARD) at time *t* i.e. the difference in the proportion of event-free individuals among those receiving A v.s B: $ARD(t)=S_{B}(t)-S_{A}(t)$ . The reciprocal of the ARD is the Number Needed To Treat (NNT) to prevent one event[22]: $NNT(t)=\frac{1}{ARD(t)}=\frac{1}{S_{B}(t)-S_{A}(t)}$
- The Hazard Ratio at time *t* i.e. the ratio of the probability of experiencing an event at the next point in time, knowing that the event has not occurred up to that time among those receiving A v.s. those receiving B: $HR(t)=\frac{h_{A}(t)}{h_{B}(t)}$ If proportionality of hazards is assumed, then the HR is constant for all times and may be related to the RR with the following formula: $RR(t)=\frac{1-S_{B}(t)^{HR}}{1-S_{B}(t)}$. If the HR is close to unity (e.g. in the range 0.75-1.30) then the HR will be numerically similar to the RR for sufficiently large values of the survival function (e.g. *S*(*t*)>0.50)
- The difference of RMST at time *t* among those receiving A v.s. those receiving B. This can be interpreted as the as the expected gain in event-free time over a time period of duration *t*. This is calculated as the difference in the area under two survival curves: $RMST(t)=\int_{0}^{t} S_{A}(t)dt-\int_{0}^{t} S_{B}(t)dt$ . When the time *t* tends to infinity the RMST tends to the difference in mean survival time (MST).

## A3. Simulation for the Cumulative Hazard and Survival Functions

The cumulative hazard function is defined by the following relationship: $H(t)=\int_{0}^{t} h(x)dx$ which is a non-linear function of the PGAM estimate for the *log-hazard* function. One can calculate this quantity via a straightforward simulation algorithm post-estimation:

1. Calculate the nodes (*t_j_*) and weights (*w_j_*) of a Gauss Lobatto quadrature for the integration of the hazard function: $H(t)=\int_{0}^{t} h(x)dx\cong\sum_{j=1}^{n} w_{j}h(t_{j})$. These weights are stored in the vector **w.**
2. Calculate the prediction matrix $\mathbf{X}_{\mathbf{p}}$ whose j^th^ row contains the terms of the linear functional for the log-hazard function at *t_j_*.
3. Sample one set of PGAM coefficients $\tilde{\mathbf{\psi}}_{\mathbf{k}}$ from the multivariate normal.
4. The k^th^ simulated value of the hazard function at each of the nodes is given by the column matrix: $\mathbf{h}_{k}=\exp(\mathbf{X}_{\mathbf{p}}\cdot\tilde{\mathbf{\psi}}_{\mathbf{k}})$. The j^th^ row of this matrix corresponds to the hazard function at the j^th^ node.
5. The k^th^ simulated of the cumulative hazard function at time *t* is given by the dot product: $H_{k}=\mathbf{h}_{k}\cdot\mathbf{w}$.

Steps 2-5 are repeated a large number of times (*k*=1,2,…,*K*) to generate *K* samples for *H*(*t*) which are summarized with conventional means (means, standard deviations, quantiles). One may combine this simple simulation algorithm with an adaptive Gaussian quadrature in which an initial estimate for *H_k_* e.g. obtained with the same Gauss Lobatto rule used when fitting the PGAM, is refined by doubling the order of the rule so as to check for numerical convergence. Even though this was not required in the datasets we analyzed, one may have to resort to adaptive Gaussian quadrature when the sampled $\tilde{\mathbf{\psi}}_{\mathbf{k}}$comes from the tail of a multivariate normal with an ill-conditioned covariance matrix. In such a case integration of the hazard may require substantially more nodes than the one used during model fitting.

Once has obtained predictions of the cumulative hazard function, then it is straightforward to calculate the corresponding value for the survival function, *S*(*t*), using the well known relationship between these two functions. In particular the k^th^ simulated value of *S_k_* is given by $S_{k}=\exp(-H_{k})$. By obtained the survival probabilities at a large number of times, one may obtain the restricted mean survival time by computing the corresponding area under the curve using any numerical integration scheme. By calculating the survival probabilities in the two arms of a study, one may form their ratios and differences so as to calculate the relative and absolute risk reductions. Since the computationally intensive steps 3-6 of the simulation are independent, one may generate the required samples and calculated these non-linear functions with “embarrassingly” parallel algorithms[23,24] distributed over heterogeneous computing platforms (multi-core processors, clusters and even specialized graphics cards).

**A4. References**

1. Cox DR. Regression Models and Life-Tables. Journal of the Royal Statistical Society Series B (Methodological). 1972;34: 187–220.

2. Nelder JA, Wedderburn RWM. Generalized Linear Models. Journal of the Royal Statistical Society Series A (General). 1972;135: 370–384.

3. Breslow N. Covariance analysis of censored survival data. Biometrics. 1974;30: 89–99.

4. Brown CC. On the Use of Indicator Variables for Studying the Time-Dependence of Parameters in a Response-Time Model. Biometrics. 1975;31: 863–872.

5. Mantel N, Hankey BF. A logistic regression analysis of response-time data where the hazard function is time dependent. Communications in Statistics - Theory and Methods. 1978;7: 333. doi:10.1080/03610927808827627

6. Thompson WA. On the treatment of grouped observations in life studies. Biometrics. 1977;33: 463–470.

7. Breslow N. Contribution to the discussion on the paper of D.R. Cox : “Regression Models and Life-Tables.” Journal of the Royal Statistical Society Series B (Methodological). 1972;34: 216–217.

8. Laird N, Olivier D. Covariance Analysis of Censored Survival Data Using Log-Linear Analysis Techniques. Journal of the American Statistical Association. 1981;76: 231–240.

9. Holford TR. Life tables with concomitant information. Biometrics. 1976;32: 587–597.

10. Holford TR. The analysis of rates and of survivorship using log-linear models. Biometrics. 1980;36: 299–305.

11. Aitkin M, Clayton D. The Fitting of Exponential, Weibull and Extreme Value Distributions to Complex Censored Survival Data Using GLIM. Journal of the Royal Statistical Society Series C (Applied Statistics). 1980;29: 156–163.

12. Peduzzi P, Holford T, Hardy R. A computer program for life table regression analysis with time dependent covariates. Comput Programs Biomed. 1979;9: 106–114.

13. Whitehead J. Fitting Cox’s Regression Model to Survival Data using GLIM. Journal of the Royal Statistical Society Series C (Applied Statistics). 1980;29: 268–275.

14. Lowrie EG, Laird NM, Parker TF, Sargent JA. Effect of the hemodialysis prescription of patient morbidity: report from the National Cooperative Dialysis Study. N Engl J Med. 1981;305: 1176–1181.

15. Clayton DG. Fitting a General Family of Failure-Time Distributions using GLIM. Journal of the Royal Statistical Society Series C (Applied Statistics). 1983;32: 102–109.

16. Efron B. Logistic Regression, Survival Analysis, and the Kaplan-Meier Curve. Journal of the American Statistical Association. 1988;83: 414–425.

17. Carstensen B. Demography and epidemiology: Practical use of the lexis diagram in the computer age or: Who needs the Cox model anyway. University of Copenhagen: Department of Biostatistics; 2006.

18. Ruppert D, Wand MP, Carroll RJ. Semiparametric regression during 2003–2007. Electron J Stat. 2009;3: 1193–1256. doi:10.1214/09-EJS525

19. Lambert P, Eilers PHC. Bayesian proportional hazards model with time-varying regression coefficients: a penalized Poisson regression approach. Stat Med. 2005;24: 3977–3989. doi:10.1002/sim.2396

20. Hennerfeind A, Brezger A, Fahrmeir L. Geoadditive Survival Models. Journal of the American Statistical Association. 2006;101: 1065–1075.

21. Belitz C, Brezger A, Kneib T, Lang S, Umlauf N. BayesX - Software for Bayesian inference in structured additive regression models [Internet]. 2012. Available: http://www.stat.uni-muenchen.de/~bayesx

22. Altman DG, Andersen PK. Calculating the number needed to treat for trials where the outcome is time to an event. BMJ. 1999;319: 1492–1495. doi:10.1136/bmj.319.7223.1492

23. Wilkinson B, Allen M. Parallel Programming: Techniques and Applications Using Networked Workstations and Parallel Computers. 2 edition. Upper Saddle River, NJ: Prentice Hall; 2004.

24. Delgado MS, Parmeter CF. Embarrassingly Easy Embarrassingly Parallel Processing in R. J Appl Econ. 2013;28: 1224–1230. doi:10.1002/jae.2362
